# Non-Respiratory Symptoms of Patients Infected with SARS-CoV-2 (Coronavirus Disease 2019): Lessons from Their Initial Presentation at the Hospital

**DOI:** 10.3390/medicina57040344

**Published:** 2021-04-02

**Authors:** Angelo V. Vasiliadis, Maria Tsatlidou, Dimitrios Metaxiotis, Charalampos Psomiadis, Anastasios Beletsiotis, Kostoula Arvaniti

**Affiliations:** 1 2nd Orthopaedic Department, General Hospital of Thessaloniki “Papageorgiou”, 56403 Thessaloniki, Greece; maria.tsatlidou@yahoo.com (M.T.); metaxiod@yahoo.de (D.M.); beletsiotisan@gmail.com (A.B.); 2 School of Medicine, Aristotle University of Thessaloniki, 54124 Thessaloniki, Greece; 3 Infection Control Unit, General Hospital of Thessaloniki “Papageorgiou”, 56403 Thessaloniki, Greece; mpampispsomiadis@gmail.com (C.P.); arvanitik@hotmail.com (K.A.); 4 COVID-19 Coordination Team, General Hospital of Thessaloniki “Papageorgiou”, 56403 Thessaloniki, Greece; 5 Critical Care Department, General Hospital of Thessaloniki “Papageorgiou”, 56403 Thessaloniki, Greece

**Keywords:** SARS-CoV-2, COVID-19, coronavirus, musculoskeletal system, myalgia, arthralgia

## Abstract

*Background and objectives:* As the COVID-19 pandemic spreads, it is becoming increasingly evident that this coronavirus is not limited to the respiratory system and that the musculoskeletal system can also be affected. The purpose of the present study was to describe non-respiratory symptoms of laboratory-confirmed COVID-19 cases. *Materials and Methods:* All patients with SARS-CoV-2 admitted to our hospital, between 1 August and 30 September 2020, were included in this retrospective study. Data were extracted from medical records. Epidemiological, clinical, laboratory and radiological characteristics at the initial presentation at the hospital were collected and analyzed. *Results:* A total of 79 COVID-19 patients were enrolled. The mean age of the patients was 44.08 years (age range, 18–87 years) and 59.5% were male. The most common symptoms were fatigue in 60 (75.9%) patients, followed by fever (73.4%), myalgia (51.9%), cough (41.8%), anosmia (38%) and arthralgia (36.7%). The muscles of the upper back and the knee joint were the most painful anatomic region and joint, respectively. The laboratory findings on admission showed that D-dimer, CRP and procalcitonin levels were increased, without significant gender differences (*p* > 0.05). Chest imaging demonstrated pneumonia in 20 (25.3%) patients. *Conclusions:* Our results indicate that from the onset of the symptoms of COVID-19 patients, musculoskeletal symptoms, such as fatigue, myalgia and arthralgia, were present in three-quarters of all patients. These findings could help elaborate updated triage and admission protocols for suspect COVID-19 patients at the hospital and Emergency Department presentation.

## 1. Introduction

The severe acute respiratory syndrome coronavirus 2 (SARS-CoV-2) is a highly transmissible coronavirus that emerged in late 2019 from the city of Wuhan and has caused a pandemic, named coronavirus disease 2019 (COVID-19). This coronavirus can be transmitted either by direct transmission through droplets of different sizes (e.g., coughing or sneezing) or by contact transmission through saliva and mucous membranes of the nose and eyes. At present, the COVID-19 pandemic is the defining global health crisis of our time and the greatest challenge we have faced since World War II [1,2]. These multifaceted repercussions of this pandemic are also extended to the socioeconomic and financial level and disrupt societies across the world due to devastating effects [2].

It is well established that COVID-19 infected patients will most probably present fever along with mild respiratory signs and symptoms, such as cough, shortness of breath and dyspnea [3]. Currently, there is some uncertainty about the prevalence of extra-pulmonary symptoms, such as those arising from the musculoskeletal system. Interestingly, with the progress of this coronavirus pandemic and the accumulation of scientific data, we are able to better describe the musculoskeletal symptoms of patients with COVID-19 infection [3,4]. However, there is not adequate evidence on this. The purpose of the present study was to describe non-respiratory symptoms of laboratory-confirmed COVID-19 cases. The frequency of the symptoms already present at the hospital admission could help the prompt identification of these patients, especially in the absence of classic respiratory symptoms.

## 2. Materials and Methods

### 2.1. Study Design and Participants

In this retrospective study, COVID-19 patients admitted to our hospital, between 1 August and 30 September 2020, were included. All patients had a laboratory-confirmed SARS-CoV-2 infection. The study was approved by the hospital Institutional Review Board and oral/written informed consent was obtained from patients or their relatives.

### 2.2. Data Collection

The epidemiological characteristics (including recent exposure to other COVID-19 cases, smoking status, comorbidities, orthopaedic medical history and hospitalization), clinical symptoms, laboratory and radiologic findings (plain chest radiography) were extracted from the electronic medical records. The laboratory assessments consisted of complete blood count, coagulation profile, C-reactive protein (CRP), procalcitonin (PCT) and D-dimer test. The radiologic assessments included at least a chest X-ray.

### 2.3. Laboratory Confirmation of SARS-CoV-2 Infection

The viral nucleic acid testing-based laboratory confirmation of SARS-CoV-2, performed by the hospital laboratory, was performed by real-time polymerase chain reaction (RT-PCR) according to the national recommended protocol. Nasal swabs were obtained with cotton tips rubbing the middle meatus (nasopharyngeal swabs). RNA samples from the specimens were extracted and subjected to the RT-PCR test using SARS-CoV-2 specific primers and probes.

### 2.4. Statistical Analysis

Collected data were analyzed with SPSS (Version 24.0). Continuous variables (age, days of hospitalization, blood tests) are expressed as mean and standard deviation (SD), while categorical variables (gender, smoking status, exposure history, comorbidities, orthopedic procedures, orthopedic history, hospitalization, symptoms, radiologic imaging) as percentages. The Kolmogorov–Smirnov test was utilized for normality analysis. Mann–Whitney U-test was utilized for the comparison of the quantities-continuous variables in our independent samples, for non-parametric distribution, respectively, in our population divided into two categories. Pearson-x2 (cross-tabulation) was utilized for the comparison of the categorical variables. The level of significance was set at *p* < 0.05.

## 3. Results

We enrolled 79 patients with confirmed COVID-19 diagnosis from 1 August to 30 September 2020. Table 1 summarizes the demographic and epidemiological characteristics of the patients. The mean age of the patients was 44.08 ± 17.53 years (range 18–87 years). Among them, 47 (59.5%) patients were male. More than half of the patients (54.4%) had an unclear history of exposure to other COVID-19 cases, while twenty (25.3%) patients had a work-related history of exposure. Eight (10.1%) patients had a history of an orthopedic procedure, such as total knee replacement, partial meniscectomy and tendon/ligament surgical repair, while 20 (25.3%) patients had an orthopedic medical history, such as knee osteoarthritis, lumbar disc hernia and rotator cuff tendinopathy.

**Figure 1:**
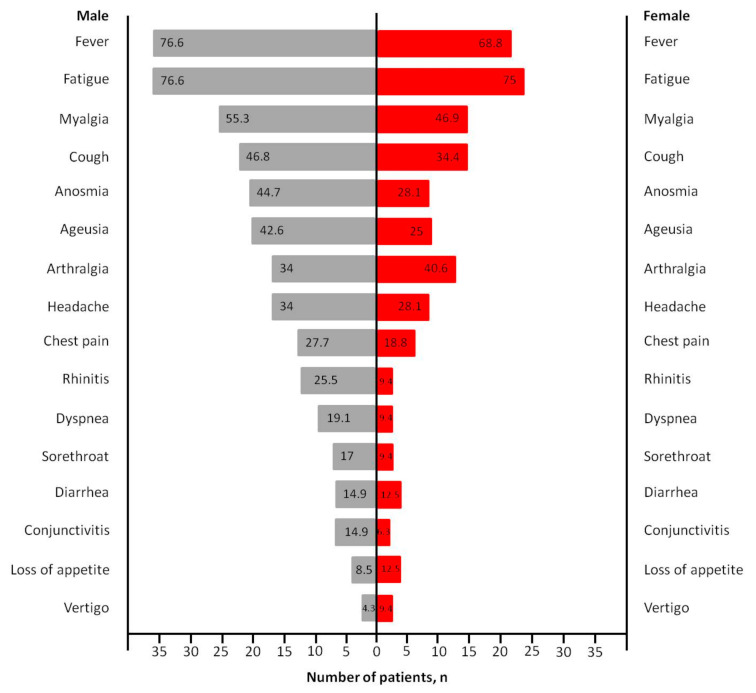
COVID-19-related general symptoms in male and female patients (numbers on the bar chart represent percentages).

**Figure 2:**
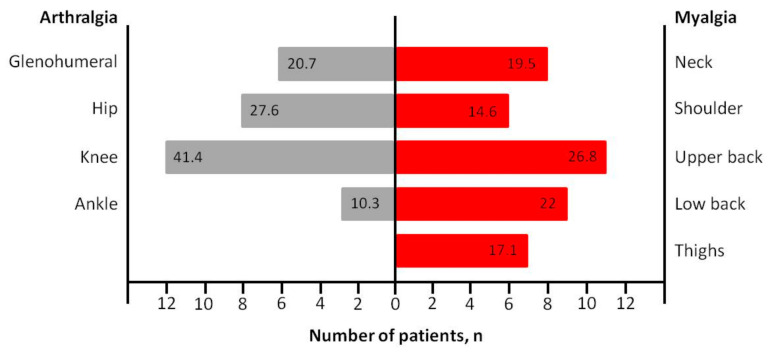
COVID-19-related musculoskeletal symptoms (numbers on the bar chart represent percentages).

The clinical characteristics of the patients are shown in Table 2. The most commonly observed symptoms at the onset of the disease manifestation were fatigue (75.9%), fever (73.4%), myalgia (51.9%) and cough (41.8%), followed by anosmia (38%), arthralgia (36.7%), ageusia (35.4%), headache (31.6%), chest pain (24.1%), rhinitis (19%), dyspnea (15.2%), sorethroat (13.9%), diarrhea (13.9%), conjunctivitis (11.4%), loss of appetite (10.1%) and vertigo (6.3%), without statistically significant differences between genders (Figure 1). Among 29 (36.7%) patients presented with arthralgia and 41 (51.9%) patients presented with myalgia, the most common painful joint was the knee involving 12 patients (41.4%) and the most common painful anatomic region was the upper back involving 11 patients (26.8%), respectively (Figure 2).

## 4. Discussion

In the present study, we identified a considerable frequency of non-respiratory symptoms, specifically musculoskeletal ones, at the initial hospital presentation of the COVID-19 cases. Fatigue, myalgia and arthralgia were present and detectable in an important part of the cases, which was not adequately emphasized at the initial scientific publications regarding the disease’s clinical manifestations. It is important to note that the elaboration of specific questionnaires focused on musculoskeletal symptoms will help clinicians to identify others who may have been exposed to COVID-19.

Currently published studies have shown that the most common onset symptoms of COVID-19 infection are fever, cough, breathing difficulty and fatigue or myalgia [1,5]. Most infected patients present with fever, while cough (81%) and fatigue (51%) also manifest at the onset of the symptoms. On the contrary, less common illness presentations include anorexia, pharyngalgia, dizziness, vomiting and diarrhea [5,6]. Those classic symptoms have been used in order to identify patients with COVID-19 infection and alert clinicians for the management of suspect cases. COVID-19 and other viral infections, such as influenza, are contagious respiratory illnesses with some similar symptoms and as a result, it may be hard to differentiate them based only on the symptoms [7,8]. These viruses have similar clinical presentations, including fever, cough, fatigue, myalgia and sometimes gastrointestinal symptoms [9]. On the contrary, COVID-19 has also demonstrated district clinical characteristics, such as anosmia and ageusia [10]. There have been several reports noting anosmia and ageusia as part of COVID-19 clinical features and this fact increases suspicion for the disease [1,7,10]. Thus, it is crucial for clinicians to distinguish COVID-19 from other respiratory infection diseases, which may mimic COVID-19 symptoms, in order to apply the proper treatment.

In the present study, fatigue was the most common symptom, affecting as many as three-quarters of COVID-19 patients. In general, viral infections can cause fatigue which is usually manifested as an overall feeling of tiredness or exhaustion. Fatigue is a common symptom in those presenting with COVID-19 infection with listed rates between 16.6% and 75% [6,11,12]. Studies have described extensive muscle dysfunction in SARS patients, while widespread muscle fiber atrophy, fiber necrosis and myofibril disarray were demonstrated in post-mortem muscle tissue of patients with SARS [4,13]. SARS-CoV-2 infection can also cause neuronal demyelination, which may further lead to additional generalized muscle weakness and fatigue [4].

According to our results, myalgia was present in as many as half of the patients (51.9%). Myalgia reflects a generalized inflammation and a cytokine response and can be the onset symptom between 16.5% and 52% of patients with COVID-19 infection [5,6,14]. Long duration of symptoms, specifically fatigue and myalgia, can be observed weeks after recovery in patients with COVID-19 infection compared with other viral infections [15]. It is known that most virus types can spread among cells extracellularly or/and intracellularly and can invade the circulatory system, causing damage to the vascular endothelium in all tissues [1,3]. Therefore, most viruses can affect all parts of the musculoskeletal system. Our findings concerning myalgia are in agreement with the rates between 44% and 52% reported by the current literature [5,16]. To the best of our knowledge, the present study demonstrated for the first time that the upper back was the most common anatomic location for myalgia in both genders, followed by low back and neck. This may be due to the fact that back pain in COVID-19 infected patients may often indicate pneumonia [15], however, further analysis is needed before conclusions are reached.

Arthralgia was reported in one-third of our patients. Patients with arthralgia were reported in 36.7% among them and it occurred most often in knees (41.4%), hip (27.6%), glenohumeral (20.7%) and ankle joint (10.3%). Until now, little is known about bone and joint disorders in patients with COVID-19 infection. Arthralgia is a serious and well-described clinical finding seen in many viral infections with a large number of causative agents reported [4,17]. However, arthralgia is often combined with myalgia, making it difficult to identify its overall prevalence [4,18]. Two studies, one single-centered and one multicenter cohort study, reported arthralgia as a unique symptom, indicating a prevalence of 2.5% in a total of 40 and 318 patients, respectively [17,19]. The majority of the studies described the presence of arthralgia, but always combined with myalgia. Zhou et al. [20] showed that 9.5% of COVID-19 infected patients complained of myalgia or arthralgia on hospital admission. In a single-centered retrospective study of 203 hospitalized patients, Chen et al. [21] observed that myalgia or arthralgia were present in 26.6% of the cases. Mo et al. [22] studied 155 patients with COVID-19 pneumonia outlining a prevalence of 61% of cases with myalgia or arthralgia. Our data showed a similarly high prevalence of arthralgia compared to the published literature from Thailand and United States [17,19].

Limitations of the study include the relatively small number of patients and the short duration of the study, although our study is meaningful because musculoskeletal symptoms were not extensively described in the recent literature. The findings of our study also offer new and potentially useful information for this patient population. Further analysis could improve the generalization of the study results. 

## 5. Conclusions

In conclusion, in our single-centered cohort study, fatigue is the most common symptom, reported by at least 75% of the patients. Approximately half of the COVID-19 patients reported myalgia, with the upper back being the most common anatomical location. In addition, arthralgia was reported in one-third of the COVID-19 patients and the knee was the most affected joint. Elaboration of questionnaires and checklists including the search of musculoskeletal symptoms (besides the classic respiratory symptoms) could help the prompt identification of suspect COVID-19 patients and the triage procedures at their initial hospital or Emergency Department presentation.

## Figures and Tables

**Table 1:** Demographic and epidemiological characteristics of patients infected with SARS-CoV-2 (COVID-19).

	All (*n* = 79)	Male (*n* = 47)	Female (*n* = 32)	*p*-Value
Age	44.08 ± 17.53	42.21 ± 16.76	46.81 ± 18.52	0.353
Smoking status				0.497
None	29 (36.7)	17 (36.2)	12 (37.5)	
Active	48 (60.8)	28 (59.6)	20 (62.5)	
Former	2 (2.5)	2 (4.2)		
Exposure history				0.501
Family cluster	16 (20.3)	9 (19.1)	7 (21.9)	
Work related	20 (25.3)	10 (21.3)	10 (31.2)	
Other	43 (54.4)	28 (59.6)	15 (46.9)	
Co-morbidities				
All	30 (38)	17 (36.2)	13 (40.6)	0.653
Hypertension	22 (27.8)	13 (27.7)	9 (28.1)	0.964
Cardiovascular disease	10 (12.7)	4 (8.5)	6 (18.8)	0.179
Diabetes Mellitus	5 (6.3)	4 (8.5)	1 (3.1)	0.334
Hypothyroidism	5 (6.3)	3 (6.4)	2 (6.2)	0.981
Dislipidemia	7 (8.9)	4 (8.5)	3 (9.3)	0.894
Asthma	3 (3.8)	1 (2.1)	2 (6.2)	0.406
Depression	5 (6.3)	3 (6.4)	2 (6.2)	0.981
GERD	4 (5.1)	2 (4.3)	2 (6.2)	0.691
Orthopaedic procedures	8 (10.1)	6 (12.8)	2 (6.2)	0.346
Orthopaedic history	20 (25.3)	10 (21.3)	10 (31.3)	0.317
Hospitalization	23 (29.1)	14 (29.8)	9 (28.1)	0.873
Days of hospitalization	13.26 ± 7.89	11.64 ± 7.49	15.78 ± 8.27	0.336

Abbreviations: GERD, gastroesophageal reflux disease.

**Table 2:** Clinical characteristics, selected laboratory findings and radiologic imaging (plain chest X-ray) of patients infected with SARS-CoV-2 (COVID-19).

	All (*n* = 79)	Male (*n* = 47)	Female (*n* = 32)	*p*-Value
Symptoms				
Arthralgia	29 (36.7)	16 (34)	13 (40.6)	0.551
Myalgia	41 (51.9)	26 (55.3)	15 (46.9)	0.461
Fatigue	60 (75.9)	36 (76.6)	24 (75)	0.871
Fever	58 (73.4)	36 (76.6)	22 (68.8)	0.438
Chest pain	19 (24.1)	13 (27.7)	6 (18.8)	0.363
Cough	33 (41.8)	22 (46.8)	11 (34.4)	0.271
Anosmia	30 (38)	21 (44.7)	9 (28.1)	0.137
Ageusia	28 (35.4)	20 (42.6)	8 (25)	0.109
Headache	25 (31.6)	16 (34)	9 (28.1)	0.579
Blood tests				
WBC	6711.8 ± 2983.5	7228.5 ± 3399.4	6096.7 ± 2330	0.326
Hb	13.9 ± 1.5	14.3 ± 1.2	13.3 ± 1.6	0.031
Hct	40.9 ± 5.2	42.1 ± 5.8	39.5 ± 4	0.006
INR	1.08 ± 0.09	1.07 ± 0.07	1.08 ± 0.12	0.982
CRP	6.2 ± 4.3	6.7 ± 4.02	5.6 ± 4.6	0.155
Procalcitonin	0.22 ± 0.27	0.22 ± 0.31	0.22 ± 0.23	0.755
D-Dimers	922.9 ± 776.4	940 ± 649.2	902.5 ± 921.8	0.480
Radiologic imaging (X-ray)				
Normal	59 (74.7)	32 (68.1)	27 (84.4)	0.239
Unilateral pneumonia	15 (19)	12 (25.5)	3 (9.4)	
Bilateral pneumonia	5 (6.3)	3 (6.4)	2 (6.2)	

Abbreviations: WBC—white blood cells; Hb—hemoglobin; Hct—hematocrit; INR—international normalized ratio; CRP—c-reactive protein.

## Data Availability

The data presented in this study are available on request from the corresponding author.
